# Intestinal Dysbiosis in Young Cystic Fibrosis Rabbits

**DOI:** 10.3390/jpm11020132

**Published:** 2021-02-16

**Authors:** Xiubin Liang, Mohamad Bouhamdan, Xia Hou, Kezhong Zhang, Jun Song, Ke Hao, Jian-Ping Jin, Zhongyang Zhang, Jie Xu

**Affiliations:** 1 Center for Advanced Models for Translational Sciences and Therapeutics, University of Michigan Medical Center, University of Michigan Medical School, Ann Arbor, MI 48109, USA; lixiubin@med.umich.edu (X.L.); songjun@med.umich.edu (J.S.); 2 Department of Physiology, Wayne State University School of Medicine, Detroit, MI 48201, USA; mbouhamdan@gmail.com (M.B.); hou_xia@hotmail.com (X.H.); jjin@med.wayne.edu (J.-P.J.); 3 Center for Molecular Medicine and Genetics, Wayne State University School of Medicine, Detroit, MI 48201, USA; kzhang@med.wayne.edu; 4 Department of Genetics and Genomic Sciences, Icahn School of Medicine at Mount Sinai, New York, NY 10029, USA; ke.hao@mssm.edu

**Keywords:** cystic fibrosis, rabbits, intestinal dysbiosis, feces microbiome

## Abstract

Individuals with cystic fibrosis (CF) often experience gastrointestinal (GI) abnormalities. In recent years, the intestinal microbiome has been postulated as a contributor to the development of CF-associated GI complications, hence representing a potential therapeutic target for treatment. We recently developed a rabbit model of CF, which is shown to manifest many human patient-like pathological changes, including intestinal obstruction. Here, we investigated the feces microbiome in young CF rabbits in the absence of antibiotics treatment. Stool samples were collected from seven- to nine-week-old CF rabbits (*n* = 7) and age-matched wild-type (WT) rabbits (*n* = 6). Microbiomes were investigated by iTag sequencing of 16S rRNA genes, and functional profiles were predicted using PICRUSt. Consistent with reports of those in pediatric CF patients, the fecal microbiomes of CF rabbits are of lower richness and diversity than that of WT rabbits, with a marked taxonomic and inferred functional dysbiosis. Our work identified a new CF animal model with the manifestation of intestinal dysbiosis phenotype. This model system may facilitate the research and development of novel treatments for CF-associated gastrointestinal diseases.

## 1. Introduction

Cystic fibrosis (CF) is an autosomal recessive disorder with a disease frequency of 1 in 2000 live births and a carrier rate of approximately 5% in the Caucasian population [1]. Mutations in the CF transmembrane conductance regulator (CFTR) gene lead to CF [2]. In 2019, the community celebrated the FDA’s approval of Trikafta, which provides benefits to greater than 90% of CF patients [3]. However, CF is not cured yet; continuous research is needed for the development of novel therapeutics for this disease. 

Clinically, CF is a progressive, chronic, and debilitating disease, affecting the lungs, sinuses, gastrointestinal (GI) tract, liver, pancreas, and others [4]. GI disease develops early and continues through adulthood in CF patients. Meconium ileus (MI) presents in up to 20% of neonates with CF, which may need surgical interventions to resolve [5]. In infancy and childhood, CF patients must be treated for pancreatic insufficiency, a condition that adversely affects intestinal nutrient absorption and subsequently weight gain and growth [6]. Constipation or distal intestinal obstruction syndrome (DIOS) often cause bloating and abdominal pain in CF patients throughout their life [7]. Furthermore, CF patients are predisposed with a 5–10 times greater risk of colorectal cancer than the general population [8]. 

Accumulating evidences show that the gut microbiome in CF patients is altered. In both pediatric and adult CF patients, their gut microbiome is of lower richness and diversity compared to those of healthy controls [9,10,11]. Such reduction of microbial diversity in CF patients is often associated with species alteration, implicating functional contributions of microbial species to CF GI diseases. However, the clinical relevance of the change in gut microorganisms is not well-established. Understanding the CF gut microbiome thus will shed light on the pathogenesis of CF GI diseases, and potentially provide hints to microbiome-based drug development. 

We recently produced CF rabbits by knocking out the CFTR gene using CRISPR/Cas9 [12]. These CF rabbits manifest many typical CF pathologies, including growth retardation, airway inflammation, and metabolic disorders, among others. Comparing to other CF animal models, CF rabbits have several advantages, for example, compared to other non-rodent models (e.g., sheep and pigs), CF rabbits are relatively cost effective to house and maintain. On the other hand, compared to the mouse model, CF rabbits are large, making many experimental procedures more practical. Importantly, rabbit airway epithelial cells responded to CFTR modulator drug VX770 in a similar manner as human airway epithelial cells do, supporting the use of these animals in preclinical studies for CF [12]. 

**Figure 1:**
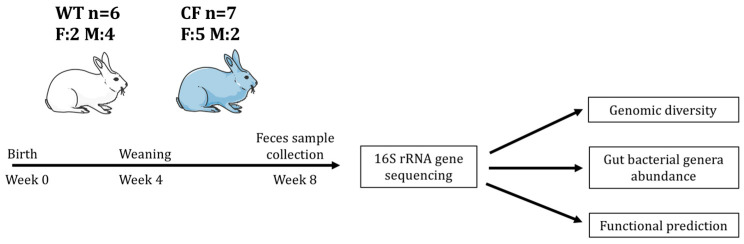
Illustration of experimental flow. F: female. M: male.

Of note, almost all CF rabbits suffer from the intestinal obstruction. In this study, we investigate the composition of feces bacteria as a proxy of gut microbiome of young CF rabbits (Figure 1). We hypothesize that the composition of bacterial communities in the CF rabbit intestine is different from that in the wild-type (WT) rabbits. In support of this hypothesis, the results revealed a marked taxonomic and inferred functional dysbiosis in the CF samples when compared to WT samples. This CF rabbit model of gut dysbiosis may facilitate the research and development of novel treatments for CF GI diseases. 

## 2. Results

### 2.1. CF Rabbits Exhibit Intestinal Obstruction

**Figure 2:**
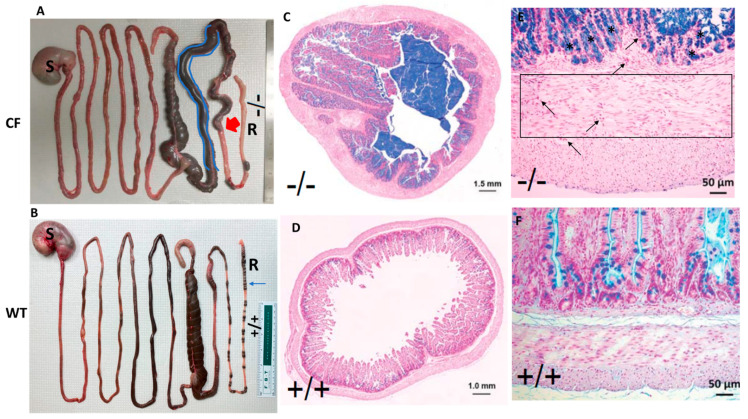
Intestinal obstruction is a common phenotype in CF rabbits. (**A**) Gross images of the GI tracks of a CF rabbit. The proximal colon (highlighted in blue) was dilated. Red arrow indicates the point of blockage. (**B**) Gross images of the GI tracks of a WT rabbit. Blue arrow indicates feces pellets, which are missing in the CF rabbit. (**C**) AB-PAS staining of the cross section of a CF rabbit colon. (**D**) AB-PAS staining of the cross section of a WT rabbit colon. (**E**) AB-PAS staining of the longitudinal sections of a CF rabbit colon. Region within the box is a representative area of interstitial fibrosis. Arrows indicate inflammatory infiltration; asterisks indicate goblet cell hyperplasia. (**F**) AB-PAS staining of the longitudinal sections of a WT rabbit colon. S: stomach. R: rectum. -/-: CFTR-/-. +/+: CFTR+/+.

Intestinal obstruction is the primary cause of mortality in CF rabbits [12], as exampled in Figure 2. The proximal colon of CF rabbits is often dilated, and the distal colon presents a paucity of stool pellets (Figure 2A), which are not observed in WT animals (Figure 2B). Interestingly, unlike CF pigs and ferrets, who need immediate surgical procedures to resolve the MI condition, many CF rabbits do not develop severe obstruction until they reach four to six weeks of age. The relatively large size of the cecum may have allowed it to accumulate feces, and hence delay the onset of the obstruction in this species. 

Alcian Blue-Periodic Acid Schiff (AB-PAS) stain of cross sections of CF rabbit colon illustrates the massive mucus plugging in the lumen (Figure 2C), but not in that of WT (Figure 2D). In longitudinal sections, the CF rabbit colon exhibits obvious intestinal wall thickening, inflammation accompanied by interstitial fibrosis, and visible goblet cell hyperplasia (Figure 2E) compared to the WT control (Figure 2F).

### 2.2. Study Sample Characteristics

We investigated the gut microbiome, surrogated by fecal samples, of CF rabbits carrying homozygous 9 base pair (bp) deletions (∆9/∆9) on the CFTR gene (*n* = 7) and WT rabbits (*n* = 6) by iTag sequencing of the 16S rRNA gene (Figure 1). After pre-processing of the sequencing data, an average of 20.7 million base pairs (Mbp) was generated to characterize the bacteria community per sample, with an average data utilization ratio of 97.6% (Supplementary Table S1). Read pairs that passed quality control (QC) were merged into consensus sequences (i.e., tags), resulting an average of 40,889 tags of good quality per sample with a mean size of 252 bp (Supplementary Table S2). The tags were clustered and mapped to 473 operational taxonomic units (OTUs). On average, each sample had 32,885 tags assigned to 220 OTUs (Table 1). The diversity of bacteria communities in fecal samples from both CF and WT rabbits was adequately captured by the sequencing effort, which is reflected by the rarefaction curves of the observed number of OTUs and Shannon Index (Supplementary Figure S1).

### 2.3. Alpha Diversity

**Figure 3:**
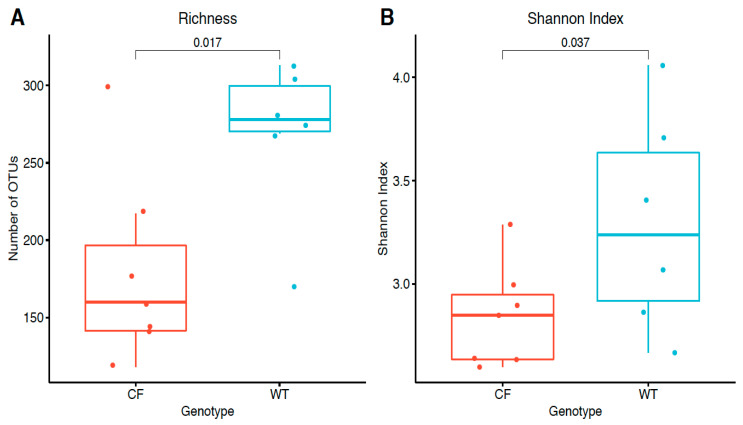
Sample alpha diversity in CF and WT groups. (**A**) Richness measured by the observed number of OTUs. (**B**) Evenness measured by the Shannon index. The distributions of values were summarized by boxplots. Shown from bottom to top is the minimum value, first quartile, median, third quartile, and maximum value, and the outlier value is shown as individual points. On top of the boxplots, the individual data points were super imposed; *p*-values were calculated by a one-sided Wilcoxon rank sum test.

We first compared the alpha diversity between the CF and WT rabbits in terms of richness, measured by the observed number of OTUs and evenness measured by the Shannon index. The CF rabbits had significantly lower bacteria richness (mean difference: −90; *p* = 0.017 in a Wilcoxon one-sided test) and Shannon index (mean difference: −0.45; *p* = 0.037 in a Wilcoxon one-sided test) (Figure 3), suggesting attenuated bacteria diversity (richness and evenness) in the fecal samples of the CF rabbit model.

### 2.4. Beta Diversity

**Figure 4:**
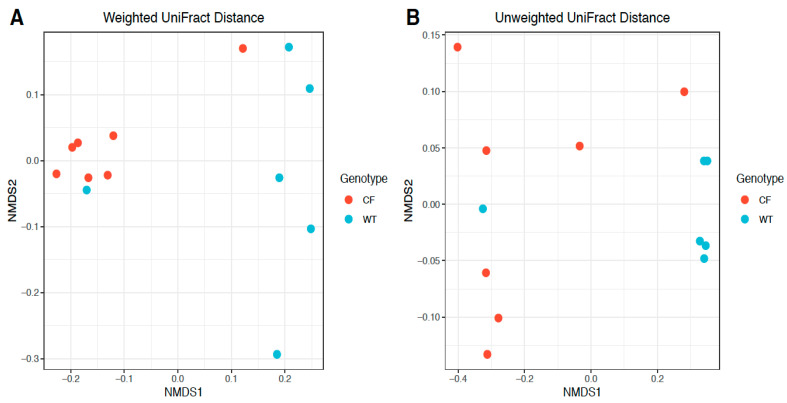
Beta diversity comparison between CF and WT groups based on OTU abundance. Beta diversity was calculated by phylogeny-based (**A**) weighted and (**B**) unweighted UniFrac distances based on relative abundances of identified OTUs, and visualized by non-metric multidimensional scaling (NMDS).

Phylogeny-based beta diversity calculated by weighted and unweighted UniFrac distances [13] was visualized by non-metric multidimensional scaling (NMDS) [14] and compared between CF and WT rabbits. Figure 4 shows a clear distinction of bacteria communities in CF rabbits from those in WT rabbits. A significant difference between the CF and WT bacteria communities was also present by PERMANOVA (permutational multivariate analysis of variance) tests (weighted UniFrac distance *R*2 = 0.38, *p* = 0.007; unweighted UniFrac distance *R*2 = 0.33, *p* = 0.016), further highlighting the difference in the composition of bacteria between CF and WT rabbits (Figure 4).

### 2.5. Relative Abundance of Bacterial Genera in CF and WT Rabbits

**Figure 5:**
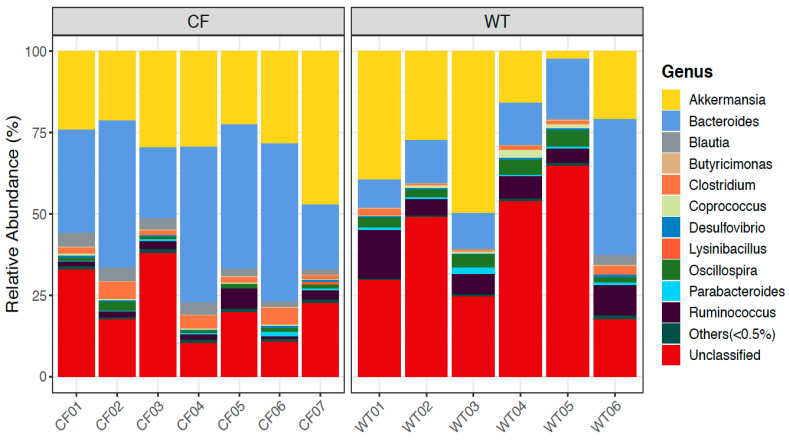
Relative abundance of bacterial genera in CF and WT rabbits.

We further looked into the composition of bacteria communities at different taxonomical ranks, and compared them between CF and WT groups. Among the top abundant genera in rabbits (Figure 5), *Bacteroides*, *Ruminococcus*, *Blautia*, and *Parabacteroides* also appeared highly abundant in humans, while other genera, such as *Akkermansia*, *Clostridium*, *Coprococcus*, and *Oscillospira*, were present uniquely in rabbits (11). Bacteria taxa at the phylum and genus level with a significantly differential abundance (at FDR <= 0.1) between CF and WT groups are reported in Table 2. More results of differentially abundant taxa at other ranks are reported in Supplementary Table S3. In the fecal samples, CF rabbits had more *Bacteroidetes* and less *Firmicutes*, *Saccharibacteria*, and *Cyanobacteria* at the rank of phylum than WT rabbits. At the genus level, *Bacteroides*, *Blautia*, and *Holdemania* were more abundant, whereas *Oscillospira*, *Roseburia*, *Ruminococcus*, and *Dehalobacterium* were less abundant in the CF rabbit model.

### 2.6. Predicted Functional Analysis by PICRUSt

We used PICRUSt [15] to predict the functional profiles of bacteria communities in the fecal samples of the rabbits based on 16S rRNA data, and compared the relative abundance of predicted KEGG orthology (KO) terms between the CF and WT groups (see details in Section 4.7). In total, relative abundances (in percentages) of 76 KO terms were predicted, and 18 of them had differential abundances between CF and WT rabbits at FDR <= 0.1 (Table 3). In particular, biological functions, such as short chain fatty acid (SCFAs; e.g., propanoate and butanoate) metabolism, lipid biosynthesis, bacterial motility, and chemotaxis were downregulated, while amino acid metabolism, lipopolysaccharide biosynthesis, and glycan degradation were upregulated in CF rabbits compared to WT rabbits.

## 3. Discussion

Recent studies have demonstrated altered intestinal microbiomes in CF patients across different age groups compared to those in healthy individuals. In infant CF patients, Antosca et al. reported reduced levels of *Bacterioides*, a bacterial genus associated with immune modulation, in their fecal microbiome [10]. In juvenile and young adult CF subjects aged 10–22 years, Miragoli et al. reported a lower frequency of sulfate-reducing bacteria in their fecal samples, which may have contributed to the abdominal bloating in CF patients [9]. In another study on CF children aged between 0.8 to 18 years, CF fecal samples exhibited marked taxonomic and functional changes of the gut microbiome [11]. These findings suggest that the gut microbiome plays an important role in CF-associated GI disease development, likely in an age-dependent manner. 

In the present work, we report a novel CF animal model (i.e., the CF rabbits) that manifests intestinal dysbiosis. Preclinical animal models are a prerequisite to test therapeutic strategies targeting the gut microbiomes of CF patients. To date, almost all CF animal gut microbiome studies have used mice [16,17,18]. Interestingly, loss of functional CFTR in CF mice is associated with significant decreases in GI bacterial community richness, evenness, and diversity, and reduced relative abundance of putative protective species in some reports [16], but not in others [17]. In the other study utilizing a nonmurine model ferrets, *Streptococcus* and *Escherichia coli* were more abundant in the CF animals than in the non-CF controls; however, it is not known whether there is a reduction of bacterial diversity due to the CF condition in these animals [19]. There are no reports from other CF animals, such as rats, pigs, and sheep. The addition of CF rabbits to the gut microbiome toolbox therefore provides a new system to the research community, and is expected to facilitate study on the pathogenesis of CF-associated GI disease and accelerate the development of novel treatments for CF gastrointestinal diseases. For example, taurine conjugate ursodeoxycholic acid (TUDCA) is being tested to treat CF-related liver disease (CFLD) (Available online: ClinicalTrials.gov ID#: NCT00004441 (accessed on 15 February 2021)). Whether TIDCA treatment alters the CF intestinal microbiome is an intriguing question, which can be tested in the CF rabbits. 

Consistent with reports of those in pediatric CF patients, the fecal microbiome of CF rabbits, in comparison to that of WT rabbits, is of lower richness and diversity, with marked taxonomic and functional alterations. At the phylum level in the fecal microbiome, *Firmicutes* (↓17%) and *Bacteroidetes* (↑17%) are the two most changed in CF conditions, albeit at different directions (Table 2). The Firmicutes/Bacteroidetes (F/B) ratio is postulated as an indicator of nutritional intake status [20]. In obese individuals, this ratio is reported to be higher than that of healthy control individuals, whereas dietary restriction led to a reduction of this ratio [20]. In the young rabbits of the current study, the F/B ratio was greatly reduced in the CF fecal microbiome in comparison to that of WT, which indicates a nutritional insufficiency due to CFTR deficiency. Future studies should test whether modulating the F/B ratio can improve the nutritional status of CF individuals. 

At the genus level, *Bacteroides* were increased by 19% in the CF rabbit fecal microbiome, representing the most changed species (Table 2). At the time of sample collection, rabbits were 7–9 weeks old, comparable to ~6–7 years old in human age. This observation is similar to that observed in pediatric CF individuals (aged 0.8–18 years) [11]. However, in a study of CF infants (six weeks to 12 months), the relative abundance of *Bacteroides* was consistently higher in the fecal microbiome of healthy individuals than that of the CF [10]. Our work and these studies suggest that the relative abundance of intestinal *Bacteroides* in CF subjects is age-related. 

Predicted functional analysis reveals several notable changes in biological functions (BFs) associated with the intestinal microbiome in CF rabbits (Table 3). (i) The increased abundance of microbiomes that are functionally implicated in “chaperones and folding catalysts” suggests an alteration in the endoplasmic reticulum (ER) protein folding capacity and potential activation of an ER stress response in CF rabbits. (ii) There is an upregulation of BFs of lipopolysaccharide (LPS) biosynthesis in CF rabbits compared to WT rabbits. LPS is the major component of the outer membrane of Gram-negative bacteria, and is known to induce strong innate immune responses [21]. It has been previously demonstrated that challenging the airway epithelial cells with *P. Aeruginosa* decreased the CFTR function and induced an increase in pro-inflammatory cytokines [22,23,24].In this regard, this alteration of gut microbiomes in CF individuals may have contributed to the inflammation phenotypes. (iii) Glycans are sequences of carbohydrates that are added to proteins or lipids to modulate their structure and function. Glycans modify proteins required for regulation of immune cells, and alterations have been associated with inflammatory conditions [25]. The altered microbiome abundance involved in glycan degradation also implicates the likeness of liver disease in CF rabbits, as the alteration of protein glycosylation has been observed in GI and liver diseases [25,26]. (iv) Furthermore, decreased microbiome abundance implicated in Butanoate metabolism may also be responsible for the metabolic phenotype in CF rabbits. Hepatic mitochondria are known to be the main target of the beneficial effect of butyrate-based compounds in reverting insulin resistance and fat accumulation in diet-induced obese animal models. Butyrate, produced by fermentation in the large intestine by gut microbiota, and its synthetic derivative have been demonstrated to be protective against insulin resistance and fatty livers [27]. (v) Short chain fatty acids (SCFAs) produced by gut bacteria widely participate in energy, lipid, glucose, and cholesterol metabolism in host various tissues. The *Bacteroidetes* mainly produce acetate and propionate, whereas butyrate is the primary metabolic end product of *Firmicutes* [28]. Notably, in CF subjects, BFs of short chain fatty acid metabolism and lipid biosynthesis were downregulated, but those of amino acid metabolism and glycan degradation were upregulated. These changes may have reflected the preference for different energy sources in CF rabbits at this age. 

Lastly, we should point out several limitations of the present study. First, we used fecal samples as a proxy for the intestinal microbiome. While fecal sampling is easy and non-invasive, this method has inherit disadvantages [29]; for example, it cannot provide accurate information on the spatial distribution of the microbiota along the intestine. Second, CF rabbits in this study did not receive any antibiotics, whereas antibiotics are routinely used in human patients. This factor should be considered when CF rabbits and patients’ intestinal microbiomes are compared. Third, as a routine care procedure, CF rabbits (but not the WT ones) received Golytely in the present work. A strict control using WT rabbits that also receive Golytely treatment should be included in a future study. Fourth, it should be noted that rabbits are coprophagic. While this does not affect the comparison between CF and WT rabbits, caution should be taken when comparing the rabbit data with a species that is not coprophagic. 

In summary, we investigated the intestinal microbiome in young CF rabbits. In comparison to that of WT rabbits, the CF rabbit intestinal microbiome is of lower richness and diversity, with a marked taxonomic and inferred functional dysbiosis. This model system may facilitate the research and development of novel treatments for CF gastrointestinal diseases. 

## 4. Materials and Methods

### 4.1. Animals and Fecal Sample Collection

The animal maintenance, care, and use procedures were reviewed and approved by the Institutional Animal Care and Use Committee (protocol #PRO00008218) of the University of Michigan, an AAALAC International accredited facility. All procedures were carried out in accordance with the approved guidelines.

Heterozygous CFTR∆9/WT (HT CF) rabbits were produced as described previously [12]. Male and female HT CF rabbits were bred to produce homozygous CFTR∆9/∆9, and WT rabbits were used in the present study. 

Both CF and WT rabbits were fed with Laboratory Rabbit Diet #5321 (LabDiet, St. Louis, MO, USA). At two weeks of age, the CF kits were given Golytely (an osmotic laxative, Braintree Labs, Braintree, MA, USA) by oral syringe feeding daily. The CF rabbits consumed this laxative for their entire life. 

Night fecal samples were collected on a morning when animals were at the corresponding age (Table 1) using sterile forceps into a sterile test tube. The samples were immediately put into a −80 °C freezer. 

Approximately 300 mg fecal samples/rabbit were submitted to BGI Americas Corporation (Cambridge, MA, USA) (BGI) for sample extraction, 16S/18S/ITS amplicon sequencing, and bioinformatics.

### 4.2. Histology Staining

Tissues were fixed in 10% formalin for about 24 h. Fixed specimens were embedded into paraffin blocks, cut into 5 µM sections, and stained with Alcian Blue-Periodic Acid Schiff (AB-PAS). In brief, the deparaffinized tissue sections were stained with AB solution (1 g of Alcian blue, pH = 2.5, 3 mL/L of acetic acid, and 97 mL of distilled water) for 30 min, followed by rinsing in water for 10 min, oxidizing in periodic acid (5 g/L) for 5 min, and staining with Schiff reagent as a counter stain for 10 min.

### 4.3. 16S rRNA Sequencing Data Processing

16S rRNA sequencing was conducted at BGI. Briefly, paired-end reads of 250 bp were generated with the Illumina HiSeq platform, and then were subject to the following pre-processing procedures [30]: (i) truncation of sequence reads with an average quality below 20 (on the Phred scale) over a 30 bp sliding window and removal of trimmed reads with less than 75% of their original size, as well as their paired reads; (ii) removal of reads contaminated by the adapter (15 bases overlapped by the adapter with maximal mismatch of 3 bp); and (iii) removal of reads with ambiguous bases (N base), as well as their paired reads; (iv) removal of reads with low complexity (reads with 10 consecutive repeated bases). The clean reads were de-multiplexed and assigned to corresponding samples (0 base mismatch in barcode sequences). Summary statistics for raw and processed reads are shown in Supplementary Table S1. An average of 42,460 read pairs were generated from each sample, amounting to 21.2 million bps. After cleaning with the above procedure, an average of 41,502 read pairs remained for each sample, with an average read utilization rate of 97.8%.

After removal of cleaned paired-end reads without overlaps, overlapped paired-end reads were merged into consensus sequences (i.e., tags) by FLASH [31] with the parameters: (i) overlapping length >= 15 bp; and (ii) mismatching ratio of overlapped region <= 0.1. Furthermore, primer sequences were removed from the generated tags, where the forward and reverse amplification primers were mapped to the two ends of tags, with four consecutive bases at the 3’ end of the primers being completely matched with the tags, and the mismatch bases of the remaining primer being no more than two. On average, 40,889 tags were generated per sample after removal of primer sequences, and the average length was 252 bp (Supplementary Table S2).

The generated tags were further clustered to operational taxonomic units (OTUs) by USEARCH (v7.0.1090) [32], detailed as follows: (i) the tags were clustered into OTUs with a 97% threshold using UPARSE [33], and the representative sequence for each OTU was derived; (ii) chimeras were identified and filtered by UCHIME (v4.2.40) [34], with the 16S rRNA sequences being screened against the “Gold” database (v20110519); (iii) the tags were mapped to OTU representative sequences using USEARCH (usearch_global command), and the number of tags mapped to each OTU in each sample was quantified as the abundance of OTUs; and (iv) OTU representative sequences were taxonomically classified by the Ribosomal Database Project (RDP) Classifier (v.2.2) [35] trained on the Greengenes database (version gg_13_5) [36] using a 0.6 confidence cutoff. OTUs that were not assigned to a bacteria taxonomical term were excluded from downstream analysis. A total of 427,508 tags from the 13 samples were clustered and mapped to 473 OTUs, none of which was singleton.

### 4.4. Alpha Diversity Analysis

The alpha diversity indices, including the observed number of OTUs and Shannon index, were calculated by Mothur (v1.31.2) [37]. The corresponding rarefaction curves were calculated based on OTUs derived from randomly extracted tags in an incremental step of 500 (Supplementary Figure S1).

### 4.5. Beta Diversity Analysis

Beta diversity was evaluated by phylogeny-based weighted and unweighted UniFrac distances, which take into account the distance of evolution between species to compare the composition of the bacteria community between samples [13]. Beta diversity analysis was performed by QIIME (v1.80) [38]. In the analysis, sequences (tags) were randomly sampled according to the minimum sequence number across all samples in order to account for the differences in the sequencing depth of different samples, and the abundance of OTUs was then adjusted accordingly. Weighted and unweighted UniFrac distances between samples were visualized by non-metric multidimensional scaling (NMDS), implemented by the R function isoMDS [14]. Permutational multivariate analysis of variance (PERMANOVA) tests were used to derive the significance level of the difference in beta diversity measurements between CF and WT groups, which was implemented by the function adonis in the R package vegan (v2.5-6) [39].

### 4.6. Differential Abundance Analysis

Metastats [40] was employed to identify differentially abundant taxa between CF and WT groups at various taxonomical ranks (phylum, class, order, family, genus, and species). The *p*-values generated at each taxonomical rank were, respectively, adjusted by a Benjamini–Hochberg false discovery rate (FDR) correction [41]. Significant differences were defined at FDR <= 0.1.

### 4.7. Functional Analysis

We used PICRUSt [15] to predict the functional profiles of the fecal bacteria communities in the CF and WT rabbit models based on 16S rRNA data. PICRUSt uses phylogenetic modeling to predict the metagenome of a microbiome community based on 16S rRNA data and reference microbiome genome databases, including Greengenes [36] and IMG [42]. The metagenome prediction results in an annotated table of predicted gene family abundance for each sample, where gene families can be functionally classified as orthologous groups in terms of KEGG orthology (KO) [43]. The relative abundances (in percentages) of predicted KO terms were compared between the CF and WT groups using a Wilcoxon rank sum test. The nominal *p*-values were adjusted by a Benjamini−Hochberg FDR correction [41]. Significant differences were defined at FDR <= 0.1.

## Figures and Tables

**Table 1:** Study sample description.

Sample ID	Genotype	Age (days)	Sex	# Tags/sample	# OTUs/sample
CF01	CF	61	F	33230	217
CF02	CF	61	F	33029	144
CF03	CF	53	F	35122	176
CF04	CF	59	F	34490	139
CF05	CF	55	M	33458	160
CF06	CF	53	M	34617	118
CF07	CF	55	F	35189	299
WT01	WT	51	M	31635	269
WT02	WT	51	F	29621	281
WT03	WT	54	M	32713	275
WT04	WT	54	M	30499	306
WT05	WT	53	F	29410	313
WT06	WT	51	M	34495	170

**Table 2:** Differentially abundant phyla and genera between CF and WT groups.

Taxon	CF Relative Abundance (%) (mean ± SD)	WT Relative Abundance (%) (mean ± SD)	Mean Difference (%) (CF − WT)	*p*-Value	FDR
**Phylum**					
Firmicutes	24.234 ± 3.975	41.307 ± 11.565	−17.073	0.002	0.023
Saccharibacteria	0.001 ± 0.003	0.081 ± 0.075	−0.08	0.010	0.035
Bacteroidetes	45.023 ± 10.483	27.355 ± 11.337	17.669	0.035	0.087
Cyanobacteria	0.008 ± 0.012	0.071 ± 0.076	−0.063	0.047	0.095
**Genus**					
Bacteroides	37.115 ± 12.404	17.673 ± 12.282	19.442	0.008	0.060
Blautia	2.997 ± 1.147	0.67 ± 1.118	2.327	0.008	0.060
Oscillospira	1.392 ± 0.718	3.517 ± 1.347	−2.125	0.005	0.060
Roseburia	0 ± 0	0.02 ± 0.023	−0.02	0.006	0.060
Ruminococcus	2.384 ± 1.836	7.777 ± 3.881	−5.392	0.008	0.060
Holdemania	0.06 ± 0.04	0.015 ± 0.015	0.045	0.014	0.086
Dehalobacterium	0.012 ± 0.029	0.08 ± 0.063	−0.069	0.017	0.089

**Table 3:** Predicted KEGG orthology terms with a significantly different abundance between CF and WT groups.

KEGG Orthology	CF Relative Abundance (%) (mean ± SD)	WT Relative Abundance (%) (mean ± SD)	Mean Difference (%) (CF − WT)	*p*-Value	FDR
Aminoacyl-tRNA biosynthesis	1.065 ± 0.032	1.117 ± 0.029	−0.053	0.002	0.056
Arginine and proline metabolism	1.361 ± 0.007	1.321 ± 0.041	0.041	0.005	0.056
Bacterial chemotaxis	0.304 ± 0.053	0.427 ± 0.102	−0.123	0.008	0.056
Chaperones and folding catalysts	1.05 ± 0.015	0.989 ± 0.042	0.061	0.005	0.056
Glycine, serine and threonine metabolism	0.873 ± 0.013	0.832 ± 0.025	0.041	0.005	0.056
Lipid biosynthesis proteins	0.634 ± 0.018	0.68 ± 0.025	−0.046	0.008	0.056
Membrane and intracellular structural molecules	0.712 ± 0.051	0.573 ± 0.088	0.14	0.008	0.056
Other glycan degradation	0.487 ± 0.052	0.356 ± 0.078	0.131	0.008	0.056
Propanoate metabolism	0.495 ± 0.017	0.568 ± 0.048	−0.073	0.008	0.056
Purine metabolism	2.062 ± 0.033	1.976 ± 0.028	0.086	0.001	0.056
Sporulation	0.439 ± 0.051	0.639 ± 0.159	−0.2	0.008	0.056
Bacterial motility proteins	0.728 ± 0.148	1.016 ± 0.198	−0.287	0.014	0.071
Butanoate metabolism	0.588 ± 0.022	0.661 ± 0.067	−0.074	0.014	0.071
Lipopolysaccharide biosynthesis proteins	0.549 ± 0.036	0.43 ± 0.099	0.119	0.014	0.071
RNA degradation	0.496 ± 0.01	0.456 ± 0.032	0.041	0.014	0.071
Flagellar assembly	0.224 ± 0.083	0.374 ± 0.13	−0.15	0.022	0.093
Mismatch repair	0.748 ± 0.017	0.781 ± 0.032	−0.033	0.022	0.093
Transcription factors	1.46 ± 0.049	1.609 ± 0.144	−0.149	0.022	0.093

## Supplementary Materials

The following are available online at https://www.mdpi.com/2075-4426/11/2/132/s1, Figure S1: Rarefaction curves for the CF and WT rabbits, Table S1: Summary statistics of processing pair-end 16S rRNA sequencing data, Table S2: Summary statistics of merged tags from pre-processed read pairs, Table S3: Differentially abundant taxa between CF and WT groups at various taxonomic ranks.
