# Supplementary material for: Intestinal Dysbiosis in Young Cystic Fibrosis Rabbits
Source: J Pers Med. 2021 Feb 16;11(2):132. doi: 10.3390/jpm11020132 (PMC7920415; doi:10.3390/jpm11020132)
Supplement: Supplementary file 1 [file jpm-11-00132-s001.zip › 04-Supplementary Materials.docx]

**Supplementary Materials**

**Supplementary Figures**

**Supplementary Figure 1. Illustration of experiment flow.**


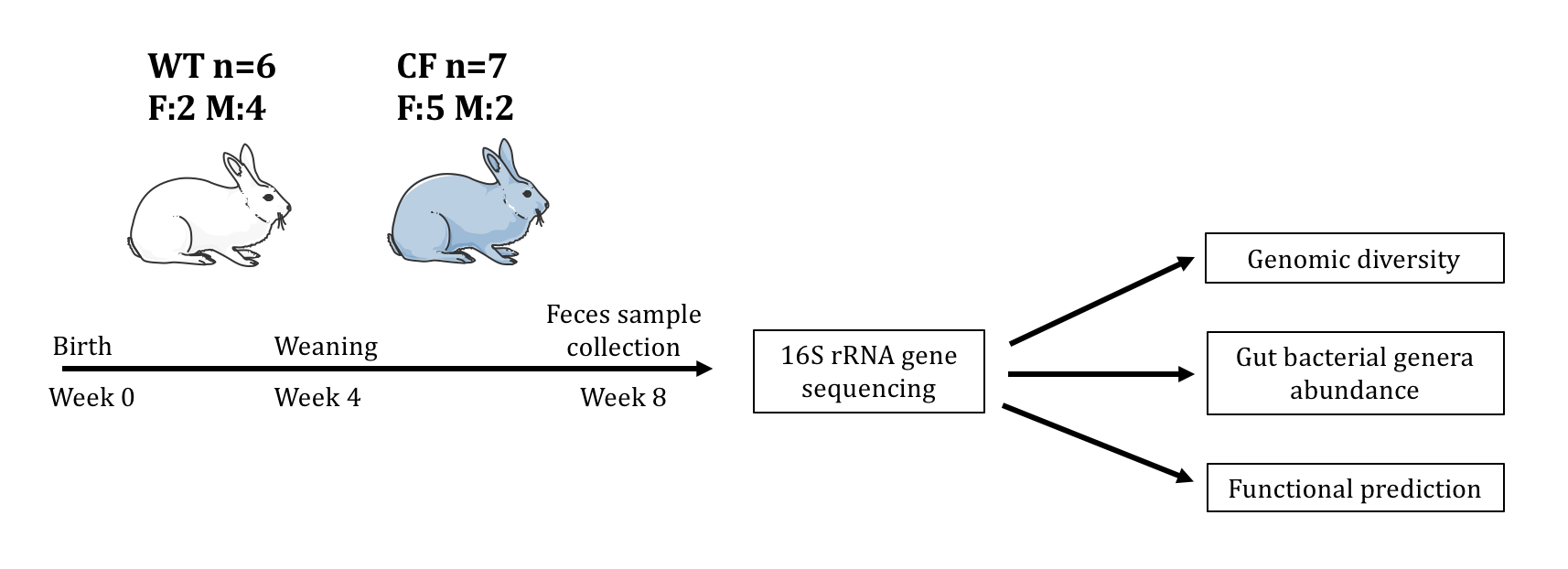


**Supplementary Figure 2. Rarefaction curves for the CF and WT rabbits.** (**A**) Observed number of OTUs; (**B**) Shannon Index.


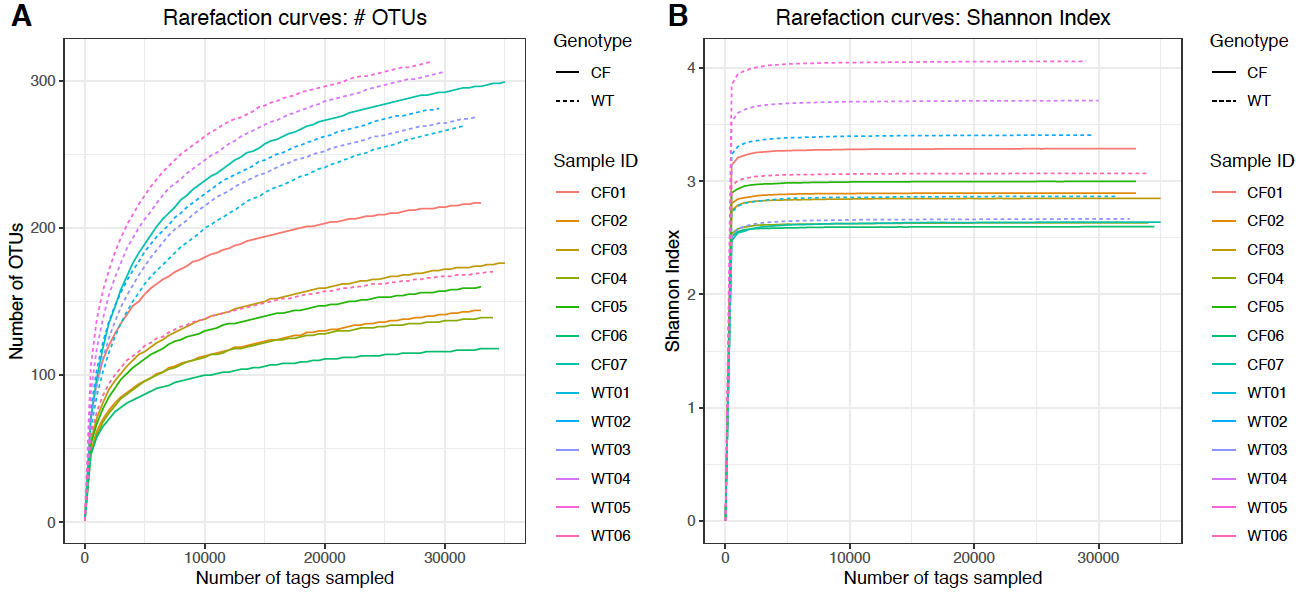


**Supplementary Tables**

**Supplementary Table 1. Summary statistics of processing pair-end 16S rRNA sequencing data.**

See Supplementary_Tables_S1_to_S3.xlsx

**Supplementary Table 2. Summary statistics of merged tags from pre-processed read pairs.**

See Supplementary_Tables_S1_to_S3.xlsx

**Supplementary Table 3. Differentially abundant taxa between CF and WT groups at various taxonomic ranks.**

See Supplementary_Tables_S1_to_S3.xlsx
